# Utility of ^18^F-Fluorodeoxyglucose Positron Emission Tomography/Computed Tomography Fusion Imaging for Prediction of Metastasis to Sentinel and Nonsentinel Nodes in Patients with Clinically Node-Negative Breast Cancer

**DOI:** 10.1245/s10434-020-08269-0

**Published:** 2020-03-02

**Authors:** Yoji Yamagishi, Tamio Yamasaki, Jiro Ishida, Tomoyuki Moriya, Takahiro Einama, Tomomi Koiwai, Makiko Fukumura-Koga, Takako Kono, Katsumi Hayashi, Hideki Ueno, Junji Yamamoto, Hitoshi Tsuda

**Affiliations:** 1 Department of Basic Pathology, National Defense Medical College, Tokorozawa, Japan; 2 Department of Surgery, National Defense Medical College, Tokorozawa, Japan; 3 Tokorozawa PET Diagnostic Imaging Clinic, Tokorozawa, Japan; 4 Sugiura Breast Gastroenterology Clinic, Tokorozawa, Japan; 5 Department of Radiology, National Defense Medical College, Tokorozawa, Japan; 6 Department of Surgery, New Tokyo Hospital, Matsudo, Japan

## Abstract

**Purpose:**

^18^F-Fluorodeoxyglucose positron emission tomography/computed tomography fusion imaging (^18^F-FDG PET/CT) is an important diagnostic tool in breast cancer. The utility of maximum standardized uptake values (SUVmax) of primary tumors has been evaluated to predict sentinel node (SN) and non-SN metastasis in clinically node-negative (cN0) patients.

**Patients and Methods:**

^18^F-FDG PET/CT was performed on 414 cN0 patients. The following parameters were evaluated: SUVmax at 60 min (SUVmax1), SUVmax at 120 min (SUVmax2), percent change between SUVmax1 and SUVmax2 (ΔSUVmax%), SN metastasis foci maximum size (SN meta size), and ratio of metastatic SNs to total SNs or SN ratio (SNR). It was assessed whether these were risk factors for SN metastasis. The relationship between these parameters and the status of SN and/or non-SN metastasis was retrospectively explored to predict non-SN metastasis.

**Results:**

All SUV parameters significantly correlated with pathological *T* factor (pT), nuclear grade, lymphatic invasion (Ly), and Ki-67 labeling index. On multivariate analysis, pT and Ly were independent predictive factors for SN metastasis. In SN meta-positive cases, SN meta size, SNR, and ΔSUVmax% were predictors for non-SN metastasis on univariate analyses, and the former two were independent predictors on multivariate analysis. The combination of SUVmax2 and ΔSUVmax% was an independent predictor of non-SN metastasis (*P* = 0.0312) and was associated with prediction of non-SN metastasis negative status with high probability (92.3%).

**Conclusions:**

In patients with cN0 breast cancer, SUV parameters of the primary tumor were correlated with pathological features. The combination of SUVmax2 and ΔSUVmax% may be useful for predicting non-SN metastasis.

**Electronic supplementary material:**

The online version of this article (10.1245/s10434-020-08269-0) contains supplementary material, which is available to authorized users.

Breast cancer is one of the most frequent malignant diseases and the fifth leading cause of cancer death in Japanese women.^1^^18^F-Fluorodeoxyglucose positron emission tomography/computed tomography fusion imaging (^18^F-FDG PET/CT) has come to play an increasing role in the diagnosis of biological properties of primary breast cancer as well as staging, treatment monitoring of residual disease, and detection of disease recurrence.^2^,^3^ Many studies have reported the correlation between the ^18^F-FDG uptake value of primary tumors and their histological and biological features such as tumor size, nuclear grade (NG), Ki-67 labeling index (LI), and prognosis.^4^–^7^ Usually, ^18^F-FDG uptake is measured with the maximum standardized uptake value (SUVmax) 60 min after its injection, but some articles report the utility of SUVmax levels both at 60 min and 120 min after injection (SUVmax1 and SUVmax2, respectively).^8^–^10^ The percentage change between SUVmax1 and SUVmax2 (ΔSUVmax%) in the primary tumor was also easily measured. However, the utility of dual time point (DTP) measurement has not yet been established for primary tumors.

Sentinel node biopsy (SNB) is a standard technique for patients with clinically node-negative (cN0) breast cancer,^11^ and axillary lymph node dissection (ALND) may be considered when macrometastasis is observed in a SN. Staging of axillary lymph node (ALN) was evaluated by physical examination and ultrasound. Nonetheless, reports of up to 30% of SN metastasis have been found in cN0 patients,^12^ and in this population, the frequency of metastasis to non-SN resected by ALND was reported to be around 40%.^13^ As the result of the American College of Surgeons Oncology Group Z0011 trial,^14^ axillary dissection in clinically node-negative individuals has come to be less common, and ALND has come to be optional for the patients who had SN-metastasis positive in two or less nodes, underwent breast-conserving surgery, and received whole-breast irradiation with adjuvant systemic therapy.

On the other hand, several nomograms were developed to predict metastasis to SN and non-SN from clinicopathological parameters, including properties of the primary tumor.^15^,^16^ The validity of these nomograms was also reported in Japanese patients.^17^ Therefore, the biological properties of the primary tumor, detected with ^18^F-FDG PET/CT, are expected to help predicting SN and/or non-SN metastasis in cN0 patients.

The aim of this study is to investigate whether the prediction of SN and non-SN metastasis is possible by the examination of SUV parameters in the primary tumor.

## Patients and Methods

### Patient Population

This study was approved by the institutional review board of the National Defense Medical College. Informed consents were obtained from all patients with regard to ^18^F-FDG-PET/CT examination and entry into this study. From September 2005 to December 2017, ^18^F-FDG-PET/CT was performed for 820 consecutive preoperative patients who received histological diagnosis of primary breast carcinoma. Of these, 406 patients were excluded from the study because of (1) preoperative medication therapy (*n* = 123), (2) ductal carcinoma in situ (DCIS) (*n* = 23), (3) distant metastasis (*n* = 5), (4) SN not being identified by SNB (*n* = 20), (5) ALND without SNB (*n* = 180), (6) difficulty measuring SUVmax (*n* = 111), (7) acquisition of one time point with ^18^F-FDG PET/CT (*n* = 7), and/or (8) diabetes mellitus (*n* = 47). These eight factors frequently overlapped. There were four cases of SN metastasis negative and non-SN metastasis positive, but these four had received preoperative medication therapy and were excluded from the study. For the 123 patients who received preoperative medication therapy, the medication was only aromatase inhibitors (AI) in 13 patients (10.6%), AI followed by tamoxifen in 1 patient (0.8%), only chemotherapy in 84 patients (68.3%), chemotherapy followed by AI in 9 patients (7.3%), chemotherapy combined antihuman epidermal growth factor receptor 2 (HER2) therapy in 14 patients (11.4%), and chemotherapy combined antiHER2 therapy followed by AI in 2 patients (1.6%). Ultimately, 414 cN0 patients were eligible for the study.

**Fig. 1:**
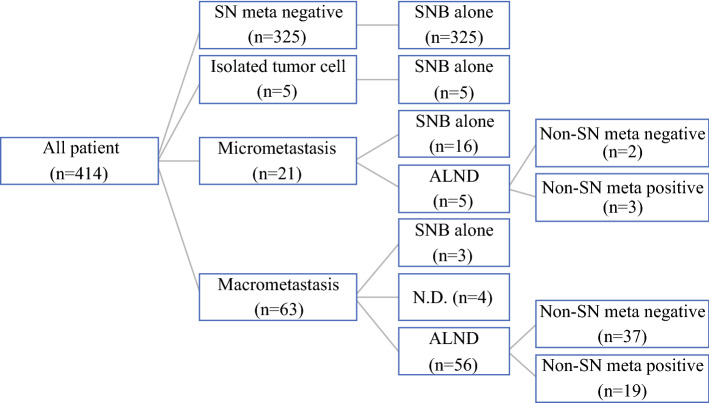
Breakdown of 414 patients with clinical node negative (cN0) breast cancer. All patients were classified into two groups with or without sentinel node (SN) metastasis (group A: SN metastasis positive; group B: negative). Fifty-six patients who received axillary lymph node dissection (ALND) with SN macrometastasis were classified into two groups with or without non-SN metastasis (group C: non-SN metastasis positive; group D: negative)

Additionally, 56 cN0 patients with SN macrometastasis and ALND were eligible (Fig. 1). ALNDs were performed for 5 of the 21 patients with micrometastasis and 56 of the 63 patients with macrometastasis. In the five patients with micrometastasis, the decisions of ALNDs were made by the surgeons during the surgery. Among the seven patients who had macrometastasis but did not receive ALNDs, three refused ALND, but the details of other four patients were unknown.

Altogether, the 414 patients had no clinical evidence of ALN metastasis by physical examination and image findings, e.g., mammography, ultrasound examination, and ^18^F-FDG PET/CT. When the axillary node status was equivocal in a patient, fine needle aspiration cytology was performed, and the case was judged cN0 if cytological examination was negative. In all these cases, the histological diagnosis of breast cancer was made by core needle biopsy before surgery. After these examinations, ^18^F-FDG PET/CT was performed prior to surgery, and the interval between core needle biopsy and surgery was 42 days on average.

## ^18^F-FDG PET/CT and Quantification of ^18^F-FDG Uptake in Primary Breast Cancer

All patients received ^18^F-FDG PET/CT scans (Biograph LSO Emotion, 3D model; Siemens, Germany) at the Tokorozawa PET Diagnostic Imaging Clinic (Tokorozawa, Japan). Patients fasted for at least 6 h before the examination. The first scan was performed 1 h after intravenous administration of 3.7 Mbq/kg ^18^F-FDG. The first scan was of whole-body images from head to thigh, and the second scan was chest only within 50–60 min after first examination.

After image reconstruction, regions of interest (ROI) were placed in one area of the primary breast lesion showing the highest ^18^F-FDG uptake. The SUV was calculated using decay-corrected tissue activity divided by the injected dose per patient body as represented by this formula

$${\text{SUV}} = {\text{activity in ROI}}\left( {\text{MBq/ml}} \right)/{\text{injected dose}}\left( {\text{MBq/kg body weight}} \right).$$

The SUVmax1 and SUVmax2 were obtained at dual time points: the SUVmax at the early (60 min) and delayed (120 min) phase, respectively. The ΔSUVmax% was calculated using the formula

$$\Delta {\text{SUVmax}}\% = \left[ {\left( {{\text{SUVmax2}} - {\text{SUVmax1}}} \right)/{\text{SUVmax1}}} \right] \times 100.$$

### Pathological Evaluation of SN

From September 2005 to March 2008, SNs were in principle identified using radioactive tin colloid alone (82 cases, 19.8%). After April 2008, SNs were in principle identified using both radioactive tin colloid and blue dye in all cases (332 cases, 80.2%). In the former era, positive rates of metastasis were 17.1% (14/82) on the patient basis and 9.8% (16/164 nodes) on the SN basis. In the latter era, positive rates of metastasis were 21.1% (70/332) on the patient basis and 15.2% (89/586 nodes) on the SN basis. For intraoperative frozen section diagnosis, each SN was sliced into 2-mm-thick pieces, cut into 5–10-µm-thick sections, fixed with formalin for a short time, and stained with hematoxylin and eosin. Tumor macrometastasis, micrometastasis, and isolated tumor cells were defined in accordance with the Union for International Cancer Control (UICC) eighth edition. For the tumor deposit size, the diameter of the largest metastatic deposit in the frozen or paraffin-embedded permanent section (maximum SN metastasis size, SN meta size) was measured. SNR was defined as numerical ratio of metastasis-positive SNs (macro- and micrometastasis) to all resected SNs.

### Histological Study

Two observers (H.T. and Y.Y.) performed pathological diagnosis. Pathological tumor size was defined as the largest diameter of a tumor including both invasive and non-invasive components, and pathological invasive tumor size was defined as the largest diameter of the invasive component of a tumor. NG was given according to the General Rules for Clinical and Pathological Recording of Breast Cancer, seventeenth edition.^18^ Estrogen receptor (ER) and progesterone receptor (PgR) were assessed by immunohistochemistry and defined as positive if 1% or higher of constituent carcinoma cells were immunoreactive.^19^ Judgment of HER2 was made according to the American Society of Clinical Oncology/College of American Pathologists guideline 2013.^20^ Ki-67 was evaluated according to the recommendation of the Breast Cancer Working Group,^21^ and Ki-67 LI was defined as high if 14% or higher of constituent carcinoma cells were immunoreactive.^22^ Pathological *T* (pT) and *N* (pN) factors and stage were determined by the clinical and pathological recording of breast cancer by UICC eighth edition.

### Memorial Sloan Kettering Cancer Center (MSKCC) Nomogram

The MSKCC nomograms were available on the MSKCC web site (http://www.mskcc.org/nomogram).^15^,^23^ The nomogram for SN metastasis required nine factors, including primary tumor features such as tumor size, grade, and lymphovascular involvement.^15^ The nomogram for non-SN metastasis required nine factors, including primary tumor features and SN status.^23^ According to the sum of points for each factor, the probabilities of SN metastasis and non-SN metastasis were calculated for each patient. The values of the probabilities were compared using the nonparametric Wilcoxon test.

### Statistical Analysis

Statistical analyses were performed using JMP^®^ 13 (SAS Institute Inc.; Cary, NC). The correlations between SUVmax parameters (SUVmax1, SUVmax2, and ΔSUVmax%) and clinicopathological factors were evaluated using the nonparametric Wilcoxon test and the Kruskal–Wallis test. Receiver operating characteristic (ROC) curves were drawn to find the optimal cutoff value of SUVmax parameters for the prediction of SN. ROC curves were also drawn to find the optimal cutoff values of SUVmax parameters, the number of SN metastasis, SNR, and SN meta size for the prediction of non-SN metastasis. The Youden index [= sensitivity − (1 − specificity) of each cutoff value] was calculated, and the highest value was taken as the optimal cutoff point. All statistical analyses were two-sided with significance defined as a *P* value of < 0.05.

## Results

### Patient Characteristics

From the 414 patients, age, cT, pathological tumor size, pathological invasive tumor size, pT, hormonal receptor status, HER2 status, Ki-67 LI, subtype, NG, Ly, histological type, pN, pStage, and SUV parameters (SUVmax1, SUVmax2, and ΔSUVmax%) were acquired (Table 1). Mean SUVmax1, SUVmax2, and ΔSUVmax% were 4.3 [± 3.2 standard deviation (SD)], 5.2 (± 4.5 SD), and 14.7 (± 20.1 SD), respectively. There was a strong correlation between SUVmax1 and SUVmax2 (*P* < 0.0001, *R*^2^ = 0.968). However, there were weak correlations between SUVmax1 and ΔSUVmax% (*P* < 0.0001, *R*^2^ = 0.184), and between SUVmax2 and ΔSUVmax% (*P* < 0.0001, *R*^2^ = 0.293).

**Table 1:** Patient characteristics

Parameter	Number	(%)
Total	414	(100.0)
Age (years)		
Mean ± SD (range)	62.4 ± 12.5	(29–91)
≥ 45	373	(90.0)
< 45	41	(10.0)
Clinical *T* factor		
cT1	248	(59.9)
cT2	158	(38.2)
cT3	8	(1.9)
Pathological tumor size (mm)		
Mean ± SD (range)	35.4 ± 21.8	(1–118)
Pathological invasive tumor size (mm)		
Mean ± SD (range)	19.4 ± 13.8	(1–90)
Pathological *T* factor		
pT1	270	(65.2)
pT2	127	(30.7)
pT3	17	(4.1)
ER		
Positive	337	(81.4)
Negative	77	(18.6)
Progesterone receptor		
Positive	308	(74.4)
Negative	106	(25.6)
HER2		
Positive	45	(10.9)
Negative	369	(89.1)
Ki-67 labeling index (%)		
Mean ± SD (range)	19.6 ± 17.0	(0–85.6)
≥ 14%	215	(51.9)
< 14%	199	(48.1)
Subtype		
ER positive/HER2 negative	312	(75.4)
ER positive/HER2 positive	25	(6.0)
ER negative/HER2 positive	20	(4.8)
ER negative/HER2 negative	57	(13.8)
Nuclear grade		
1	150	(36.2)
2	111	(26.8)
3	153	(37.0)
Lymphatic invasion		
Positive	158	(38.2)
Negative	256	(61.8)
Histological type		
Invasive ductal carcinoma	338	(81.6)
Invasive carcinoma special type	76	(18.4)
Pathological *N* factor		
pN0	330	(79.7)
pN1	72	(17.4)
pN2	10	(2.4)
pN3	2	(0.5)
Pathological stage		
I	238	(57.5)
II	157	(37.9)
III	19	(4.6)
SUVmax at 60 min		
Mean ± SD (range)	4.3 ± 3.2	(0.7–20.9)
SUVmax at 120 min		
Mean ± SD (range)	5.2 ± 4.5	(0.6–28.2)
ΔSUVmax% (%)		
Mean ± SD (range)	14.7 ± 20.1	(− 36.7–84.2)

*ER* estrogen receptor, *HER2* human epidermal growth factor receptor 2, *SD* standard deviation, *SUVmax* maximum standardized uptake value, *SUVmax1* SUVmax at 60 min, *SUVmax2* SUVmax at 120 min, *ΔSUVmax*% (SUVmax2 − SUVmax1)/SUVmax1 × 100

### Sentinel Node Status

From the 414 patients, the number of metastasis-positive SNs was 0 in 330 (79.7%), 1 in 67 (16.2%), 2 in 14 (3.4%), 3 in 2 (0.5%), and 4 in 1 (0.2%). The number of SNs removed by SNB was 1 in 198 (47.8%), 2 in 137 (33.1%), 3 in 51 (12.3%), 4 in 21 (5.1%), and 5 or more in 7 (1.7%). The SNR was 0 in 330 (79.7%), 0.13 in 1 (0.3%), 0.25 in 5 (1.2%), 0.33 in 9 (2.2%), 0.5 in 19 (4.6%), 0.67 in 8 (1.9%), and 1 in 42 (10.1%). Axillary lymph node status was classified as no metastasis in 325 (78.5%), isolated tumor cells in 5 (1.2%), micrometastasis in 21 (5.1%), and macrometastasis in 63 individuals (15.2%).

### Comparison Between SN Metastasis-Positive and Metastasis-Negative Groups

The number of patients with SN metastasis, including macrometastasis and micrometastasis, was 84 (20.3%) (Fig. 1). All patients were classified into either SN-metastasis positive (group A) or SN-metastasis negative (group B). Clinicopathological factors were compared between the two, and results are presented in Table 2. There were significant differences between the groups in cT (cT1, cT2 versus cT3) (*P* = 0.0103), the mean pathological tumor size (*P* = 0.0017), the mean pathological invasive tumor size (*P* < 0.0001), pT (*P* < 0.0001), ER (*P* = 0.0070), PgR (*P* = 0.0031), and Ly (*P* < 0.0001). With regard to HER2, Ki-67 LI, and NG, there were no significant differences between the two groups.

**Table 2:** Comparison of clinicopathological parameters between SN metastasis positive (group A) and negative (group B) groups

Parameter	Total	Number of cases (%)				
		Group A		Group B		*P* value
	414	84	(20.3)	330	(79.7)	
Age (years)						
Mean ± SD (range)		62.1 ± 11.8	(39–91)	62.4 ± 12.7	(29–87)	0.591
≥ 45	373	76	(20.4)	297	(79.6)	1.000
< 45	41	8	(19.5)	33	(80.5)	
Clinical *T* factor						
cT1	248	45	(18.1)	203	(81.9)	
cT2	158	34	(21.5)	124	(78.5)	0.0103
cT3	8	5	(62.5)	3	(37.5)	
Pathological tumor size (mm)						
Mean ± SD (range)		43.0 ± 25.6	(8–107)	33.4 ± 20.3	(1–118)	0.0017
Pathological invasive tumor size (mm)						
Mean ± SD (range)		29.0 ± 20.0	(2–90)	16.9 ± 10.4	(1–70)	< 0.0001
Pathological *T* factor						
pT1	270	32	(11.9)	238	(88.1)	
pT2	127	40	(31.5)	87	(68.5)	< 0.0001
pT3	17	12	(70.6)	5	(29.4)	
Estrogen receptor						
Positive	337	77	(22.8)	260	(77.2)	0.0070
Negative	77	7	(9.1)	70	(90.9)	
Progesterone receptor						
Positive	308	73	(23.7)	235	(76.3)	0.0031
Negative	106	11	(10.4)	95	(89.6)	
Human epidermal growth factor receptor 2						
Positive	45	5	(11.1)	40	(88.9)	0.119
Negative	369	79	(21.4)	290	(78.6)	
Ki-67 labeling index (%)						
Mean ± SD (range)		19.7 ± 14.8	(0–65.2)	19.6 ± 17.5	(0–85.6)	0.331
≥ 14%	215	50	(23.3)	165	(76.7)	0.142
< 14%	199	34	(17.1)	165	(82.9)	
Nuclear grade						
1	150	27	(18.0)	123	(82.0)	0.209
2	111	19	(17.1)	92	(82.9)	
3	153	38	(24.8)	115	(75.2)	
Lymphatic invasion						
Positive	158	58	(36.7)	100	(63.3)	< 0.0001
Negative	256	26	(10.2)	230	(89.8)	
SUVmax1						
Mean ± SD (range)		4.5 ± 3.0	(0.7–13.9)	4.2 ± 3.3	(0.7–20.9)	0.149
≥ 3.4	201	49	(24.4)	152	(75.6)	0.0506
< 3.4	213	35	(16.4)	178	(83.6)	
SUVmax2						
Mean ± SD (range)		5.4 ± 4.0	(0.8–18.3)	5.2 ± 4.6	(0.6–28.2)	0.169
≥ 3.0	249	59	(23.7)	190	(76.3)	0.0348
< 3.0	165	25	(15.2)	140	(84.8)	
ΔSUVmax% (%)						
Mean ± SD (range)		15.2 ± 19.9	(− 36.7–72.6)	14.5 ± 20.2	(− 32.7–84.2)	0.582
≥ 2.5	299	68	(22.7)	231	(77.3)	0.0556
< 2.5	115	16	(13.9)	99	(86.1)	

*SD* standard deviation, *SUVmax* maximum standardized uptake value, *SUVmax1* SUVmax at 60 min, *SUVmax2* SUVmax at 120 min, *ΔSUVmax*% (SUVmax2 − SUVmax1)/SUVmax1 × 100

### Optimal Cutoff Values of SUVmax Parameters for Prediction of SN Metastasis

The optimal cutoff values of SUVmax1, SUVmax2, and ΔSUVmax% for the prediction of SN metastasis were 3.4 [area under the curve (AUC) = 0.55, 95% confidence interval (CI) 0.48–0.62], 3.0 (AUC = 0.55, 95% CI 0.48–0.62), and 2.5 (AUC = 0.52, 95% CI 0.45–0.59), respectively.

### Univariate and Multivariate Analyses for Predictor of SN Metastasis

By univariate and multivariate logistic analyses in comparing pre- and postoperative factors, the odds ratios for SN metastasis were found to be significantly higher in the cT3 group than in the cT1/2 group, higher in the pT3 group than in the pT1/2 group, higher in the ER-positive group than in the ER-negative group, and higher in Ly-positive group than in Ly-negative group (Table 3). PgR was univariately significant but excluded from the multivariate analyses because of its collinearity with ER. Although SUVmax1 (≥ 3.4 versus < 3.4), SUVmax2 (≥ 3.0 versus < 3.0), and ΔSUVmax% (≥ 2.5 versus < 2.5) were also correlated with the risk of SN metastasis in the univariate analyses, these factors were not significant in the multivariate analyses (Table 3A, B). SUVmax1, SUVmax2, and ΔSUVmax% were correlated with each other. Additionally, multivariate analyses were conducted incorporating parameters that are available preoperatively, i.e., cT, ER, and SUVmax parameters, which revealed that cT, ER, and SUVmax2 were significant in one of the multivariate analyses (Table 3C).

**Table 3:** Univariate and multivariate logistic model analyses for odds estimation of SN metastasis (*n* = 414)

Parameter	Unfavorable	Favorable	Odds ratio	95% CI	*P* value
*A Univariate analyses*					
Age (years)	≥ 45	< 45	1.06	0.47–2.38	0.896
Clinical *T* factor	cT3	cT1, 2	6.90	1.61–29.5	0.0091
Pathological *T* factor	pT3	pT1, 2	10.8	3.70–31.7	< 0.0001
Estrogen receptor	Positive	Negative	2.96	1.31–6.71	0.0092
Progesterone receptor	Positive	Negative	2.68	1.36–5.28	0.0043
HER2	Negative	Positive	2.18	0.83–5.71	0.113
Ki-67 labeling index (%)	≥ 14%	< 14%	1.47	0.90–2.39	0.120
Nuclear grade	3	1, 2	1.54	0.95–2.51	0.0794
Lymphatic invasion	Positive	Negative	5.13	3.05–8.62	< 0.0001
SUVmax1	≥ 3.4	< 3.4	1.64	1.01–2.66	0.0456
SUVmax2	≥ 3.0	< 3.0	1.74	1.04–2.91	0.0356
ΔSUVmax% (%)	≥ 2.5	< 2.5	1.82	1.01–3.30	0.0476

Parameter (unfavorable versus favorable)	Odds ratio	95% CI	*P* value	Odds ratio	95% CI	*P* value	Odds ratio	95% CI	*P* value
*B Multivariate analyses including postoperative factors, and SUVmax1, SUVmax2, or ΔSUVmax*%									
Pathological *T* factor (pT3 versus pT1, pT2)	9.37	2.85–30.8	0.0002	9.62	2.92–31.7	0.0002	9.94	3.06–32.4	0.0001
Estrogen receptor (positive versus negative)	3.75	1.54–9.16	0.0036	3.69	1.52–8.96	0.0040	3.63	1.50–8.82	0.0043
Lymphatic invasion (positive versus negative)	5.02	2.91–8.64	< 0.0001	5.00	2.90–8.64	< 0.0001	4.97	2.87–8.63	< 0.0001
SUVmax1 (≥ 3.4 versus < 3.4)	1.29	0.75–2.22	0.354						
SUVmax2 (≥ 3.0 versus < 3.0)				1.21	0.69–2.14	0.503			
ΔSUVmax% (%) (≥ 2.5 versus < 2.5)							1.21	0.63–2.32	0.565
*C Multivariate analyses including preoperative factors, and SUVmax1, SUVmax2, or ΔSUVmax*%									
Clinical *T* factor (cT3 versus cT1, cT2)	5.72	1.29–25.4	0.0217	6.00	1.36–26.5	0.0180	5.93	1.34–26.1	0.0187
Estrogen receptor (positive versus negative)	3.14	1.37–7.19	0.0068	3.10	1.36–7.11	0.0073	3.03	1.33–6.93	0.0085
SUVmax1 (≥ 3.4 versus < 3.4)	1.64	0.99–2.71	0.0504						
SUVmax2 (≥ 3.0 versus < 3.0)				1.74	1.03–2.96	0.0376			
ΔSUVmax% (%) (≥ 2.5 versus < 2.5)							1.77	0.97–3.23	0.0628

*CI* confidence interval, *HER2* human epidermal growth factor receptor 2, *SN* sentinel node, *SUVmax* maximum standardized uptake value, *SUVmax1* SUVmax at 60 min, *SUVmax2* SUVmax at 120 min, *ΔSUVmax*% (SUVmax2 − SUVmax1)/SUVmax1 × 100

### Prediction of Non-SN Metastasis in Patients with SN Macrometastasis

Among 63 patients with SN macrometastasis, the 56 patients who received ALND were eligible (Fig. 1). These patients were classified into two groups with or without nonmetastasis (group C and D). Group C was non-SN metastasis positive (*n* = 19, 33.9%), and group D was non-SN metastasis negative (*n* = 37, 66.1%).

### Optimal Cutoff Values of SN Status and SUVmax Parameters for Prediction to Non-SN Metastasis

The optimal cutoff values of number of SN metastases, SNR, and SN meta size for the prediction of non-SN metastasis were 2.0 (AUC = 0.55, 95% CI 0.42–0.68), 0.67 (AUC = 0.63, 95% CI 0.50–0.76), and 6.0 mm (AUC = 0.72, 95% CI 0.57–0.87), respectively. Similarly, the optimal cutoff values of SUVmax1, SUVmax2, and ΔSUVmax% of the primary site for the prediction of non-SN metastasis were 7.6 (AUC = 0.59, 95% CI 0.43–0.75), 3.0 (AUC 0.59, 95% CI 0.43–0.75), and 20.0 (AUC = 0.57, 95% CI 0.42–0.73), respectively.

### Comparison Between Non-SN Metastasis-Positive and Metastasis-Negative Groups

The clinicopathological factors of these groups (group C and D) are presented in Table 4. There were significant differences in the mean pathological invasive tumor size (40.6 mm ± 23.3 SD versus 26.6 mm ± 19.1 SD, *P* = 0.0106), mean SN meta size (8.7 mm ± 4.2 SD versus 5.7 mm ± 3.0 SD, *P* = 0.0080), SN meta size (≥ 6.0 mm versus < 6.0 mm, *P* = 0.0111), SNR (≥ 0.67 versus < 0.67, *P* = 0.0131), and ΔSUVmax% (≥ 20.0 versus < 20.0, *P* = 0.0458). Although there was no significant difference in SUVmax1 and SUVmax2 between these two groups, they tended to be higher in group C than in group D.

**Table 4:** Comparison of clinicopathological parameters between non-SN metastasis positive (group C) and negative (group D) groups

Parameter	Total	Number of cases (%)				*P* value
		Group C	(%)	Group D	(%)	
	56	19	(33.9)	37	(66.1)	
Age (years)						
Mean ± SD (range)		61.6 ± 12.0	(41–83)	63.6 ± 11.6	(40–91)	0.672
≥ 45	52	17	(32.7)	35	(67.3)	0.598
< 45	4	2	(50.0)	2	(50.0)	
*Primary tumor feature*						
Clinical *T* factor						
cT1	32	8	(25.0)	24	(75.0)	0.141
cT2	20	10	(50.0)	10	(50.0)	
cT3	4	1	(25.0)	3	(75.0)	
Pathological tumor size (mm)						
Mean ± SD (range)		46.2 ± 24.0	(16–90)	38.6 ± 24.3	(8–107)	0.156
Pathological invasive tumor size (mm)						
Mean ± SD (range)		40.6 ± 23.3	(11–90)	26.6 ± 19.1	(7–87)	0.0106
Pathological *T* factor						
pT1	19	4	(21.1)	15	(78.9)	0.131
pT2	27	9	(33.3)	18	(66.7)	
pT3	10	6	(60.0)	4	(40.0)	
Estrogen receptor						
Positive	50	16	(32.0)	34	(68.0)	0.397
Negative	6	3	(50.0)	3	(50.0)	
Progesterone receptor						
Positive	46	14	(30.4)	32	(69.6)	0.281
Negative	10	5	(50.0)	5	(50.0)	
Human epidermal growth factor receptor 2						
Positive	5	1	(20.0)	4	(80.0)	0.652
Negative	51	18	(35.3)	33	(64.7)	
Ki-67 labeling index (%)						
Mean ± SD (range)		24.2 ± 18.7	(1.1–65.2)	17.4 ± 13.3	(0–61.8)	0.203
≥ 14%	32	12	(37.5)	20	(62.5)	0.515
< 14%	24	7	(29.2)	17	(70.8)	
Nuclear grade						
1	18	5	(27.8)	13	(72.2)	0.806
2	10	4	(40.0)	6	(60.0)	
3	28	10	(35.7)	18	(64.3)	
Lymphatic invasion						
Positive	38	14	(36.8)	24	(63.2)	0.503
Negative	18	5	(27.8)	13	(72.2)	
*Sentinel node feature*						
Number of SN metastasis						
≥ 2	16	7	(43.7)	9	(56.3)	0.326
< 2	40	12	(30.0)	28	(70.0)	
SN meta size (mm)						
Mean ± SD (range)		8.7 ± 4.2	(2.3–18.0)	5.7 ± 3.0	(2.1–11.0)	0.0080
≥ 6 mm	28	14	(50.0)	14	(50.0)	0.0111
< 6 mm	28	5	(17.9)	23	(82.1)	
SN ratio						
≥ 0.67	38	17	(44.7)	21	(55.3)	0.0131
< 0.67	18	2	(11.1)	16	(88.9)	
*SUV parameters*						
SUVmax1						
Mean ± SD (range)		5.4 ± 3.5	(1.1–13.9)	4.4 ± 2.9	(0.7–12.8)	0.279
≥ 7.6	11	6	(54.5)	5	(45.5)	0.107
< 7.6	45	13	(28.9)	32	(71.1)	
SUVmax2						
Mean ± SD (range)		6.5 ± 4.6	(0.8–17.6)	5.3 ± 4.0	(0.8–18.3)	0.283
≥ 3.0	41	17	(41.5)	24	(58.5)	0.0612
< 3.0	15	2	(13.3)	13	(86.7)	
ΔSUVmax% (%)						
Mean ± SD (range)		16.9 ± 15.3	(− 27.7–45.7)	13.8 ± 20.6	(− 36.7–53.6)	0.382
≥ 20.0	25	12	(48.0)	13	(52.0)	0.0458
< 20.0	31	7	(22.6)	24	(77.4)	

*SD* standard deviation, *SN* sentinel node, *SN meta size* maximum sentinel node metastasis size, *SN ratio* number of metastasis-positive SNs/number of all resected SNs, *SUVmax* maximum standardized uptake value, *SUVmax1* SUVmax at 60 min, *SUVmax2* SUVmax at 120 min, *ΔSUVmax*% (SUVmax2 − SUVmax1)/SUVmax1 × 100

### Univariate and Multivariate Analyses for Prediction of Non-SN Metastasis

On univariate analyses, SN meta size, SNR, and ΔSUVmax% were statistically significant factors for the prediction of non-SN metastasis (Table 5). On multivariate analysis, SN meta size and SNR were independent predictive factors of metastasis to non-SN in patients with SN metastasis (Table 5). ΔSUVmax% was nearly significant as a predictive factor (odds ratio 3.60, 95% CI 0.95–13.6, *P* = 0.0586).

**Table 5:** Univariate and multivariate logistic model analyses for risk factors for non-SN metastasis (*n* = 56)

Parameter	Unfavorable	Favorable	Odds ratio	95% CI	*P* value
*A Univariate analyses*					
Age (years)	< 45	≥ 45	2.06	0.27–15.9	0.489
Clinical *T* factor	cT1, 2	cT3	1.59	0.15–16.4	0.698
Pathological *T* factor	pT3	pT1, pT2	3.81	0.92–15.7	0.0647
Estrogen receptor	Negative	Positive	2.13	0.39–11.7	0.387
Progesterone receptor	Negative	Positive	2.29	0.57–9.17	0.244
HER2	Negative	Positive	2.18	0.23–21.0	0.500
Ki-67 labeling index (%)	≥ 14%	< 14%	1.46	0.47–4.53	0.515
Nuclear grade	3	1, 2	1.17	0.39–3.55	0.778
Lymphatic invasion	Positive	Negative	1.52	0.45–5.16	0.505
Number of SN metastasis	≥ 2	< 2	1.81	0.55–6.01	0.329
SN meta size	≥ 6 mm	< 6 mm	4.60	1.36–15.6	0.0141
SN ratio	≥ 0.67	< 0.67	6.48	1.30–32.2	0.0224
SUVmax1	≥ 7.6	< 7.6	2.95	0.77–11.4	0.116
SUVmax2	≥ 3.0	< 3.0	4.60	0.92–23.1	0.0636
ΔSUVmax% (%)	≥ 20.0	< 20.0	3.16	1.00–10.0	0.0498
*B Multivariate analysis*					
SN meta size	≥ 6 mm	< 6 mm	4.17	1.10–15.9	0.0367
SN ratio	≥ 0.67	< 0.67	7.88	1.34–46.3	0.0223
ΔSUVmax% (%)	≥ 20.0	< 20.0	3.60	0.95–13.6	0.0586

*CI* confidence interval, *HER2* human epidermal growth factor receptor 2, *SN* sentinel node, *SN meta size* maximum sentinel node metastasis size, *SN ratio* number of metastasis-positive SNs/number of all resected SNs, *SUVmax* maximum standardized uptake value, *SUVmax1* SUVmax at 60 min, *SUVmax2* SUVmax at 120 min, *ΔSUVmax*% (SUVmax2 − SUVmax1)/SUVmax1 × 100

### Combination of SUVmax2 and ΔSUVmax% for Prediction of Non-SN Metastasis

There were 13 patients with low SUVmax2 (< 3.0) and low ΔSUVmax% (< 20.0). Of these, 12 were patients without non-SN metastasis (92.3%) (Table 6). The sensitivity, specificity, positive predictive value, negative predictive value (NPV), and accuracy of their combination for non-SN metastasis were 94.7%, 32.4%, 41.9%, 92.3%, and 53.6%, respectively. In predicting non-SN metastasis, the combination of SUVmax2 and ΔSUVmax% showed higher sensitivity and NPV than the SUVmax1, SUVmax2, ΔSUVmax% and the combination of SUVmax1 and ΔSUVmax%. By univariate and multivariate logistic analyses, the combination of SUVmax2 and ΔSUVmax% was an independent predictive factor of metastasis to non-SN in patients with SN macrometastasis (*P* = 0.0470, 0.0312, respectively) (Table 7). However, the combination of SUVmax1 and ΔSUVmax% did not show any significant difference on univariate analysis. The combination of SUVmax2 and ΔSUVmax% was a useful predictor of metastasis to non-SN.

**Table 6:** Prediction of non-SN metastasis by SUVmax1, SUVmax2, ΔSUVmax%, and the combination SUVmax and ΔSUVmax% in primary tumor

Parameters	Number of cases			Sensitivity	Specificity	PPV	NPV	Accuracy	*P* value
	Total	Group C	Group D	(%)	(%)	(%)	(%)	(%)	
SUVmax1									
≥ 7.6	11	6	5	31.6	86.5	54.5	71.1	67.9	0.107
< 7.6	45	13	32						
SUVmax2									
≥ 3.0	41	17	24	89.5	35.1	41.5	86.7	53.6	0.0612
< 3.0	15	2	13						
ΔSUVmax% (%)									
≥ 20.0	25	12	13	63.2	64.9	48.0	77.4	64.3	0.0458
< 20.0	31	7	24						
Combination of SUVmax1 and ΔSUVmax% (%)									
SUVmax1 ≥ 7.6 and ΔSUVmax% ≥ 20.0	9	5	4	26.3	89.2	55.6	70.2	67.9	0.247
Other	47	14	33						
Combination of SUVmax2 and ΔSUVmax% (%)									
Other	43	18	25	94.7	32.4	41.9	92.3	53.6	0.0416
SUVmax2 < 3.0 and ΔSUVmax% < 20.0	13	1	12						

*NPV* negative predictive value, *PPV* positive predictive value, *SN* sentinel node, *SUVmax* maximum standardized uptake value, *SUVmax1* SUVmax at 60 min, *SUVmax2* SUVmax at 120 min, *ΔSUVmax*% (SUVmax2 − SUVmax1)/SUVmax1 × 100

**Table 7:** Univariate and multivariate logistic analyses including combination of SUVmax1, SUVmax2 and ΔSUVmax% in primary tumor for prediction of non-SN metastasis

Parameter	Unfavorable	Favorable	Odds ratio	95% CI	*P* value
*A Univariate analyses*					
SUVmax1/ΔSUVmax%	SUVmax1 ≥ 7.6 and ΔSUVmax% ≥ 20.0	Other	2.95	0.69–12.6	0.145
SUVmax2/ΔSUVmax%	Other	SUVmax2 < 3.0 and ΔSUVmax% < 20.0	8.64	1.03–72.6	0.0470
*B Multivariate analysis*					
SN meta size	≥ 6 mm	< 6 mm	4.00	1.02–15.7	0.0470
SN ratio	≥ 0.67	< 0.67	8.13	1.47–45.0	0.0165
SUVmax2/ΔSUVmax%	Other	SUVmax2 < 3.0 and ΔSUVmax% < 20.0	11.7	1.25–109.2	0.0312

*CI* confidence interval, *SN* sentinel node, *SN meta size* maximum sentinel node metastasis size, *SN ratio* number of metastasis-positive SNs/number of all resected SNs, *SUVmax* maximum standardized uptake value, *SUVmax1* SUVmax at 60 min, *SUVmax2* SUVmax at 120 min, *ΔSUVmax*% (SUVmax2 − SUVmax1)/SUVmax1 × 100

### Application of Memorial Sloan Kettering Cancer Center (MSKCC) Nomogram

According to MSKCC nomograms, the median probabilities of SN metastasis were 58.0% (11.0–98.0%) and 34.0% (0.0–93.0%) in groups A and B, respectively, and the median probabilities of non-SN metastasis were 28.0% (11.0–68.0%) and 20.0% (9.0–77.0%) in groups C and D, respectively. There were significant differences between group A and B (*P* < 0.0001) and between group C and D (*P* = 0.0296). The distributions of the probabilities of SN metastasis and non-SN metastasis are presented in Supplementary Fig. 1.

## Discussion

In the present work, the significance of SUV parameters in primary tumor for SN and/or non-SN metastasis was evaluated in patients with cN0 breast cancer. SUV parameters were found to be effective predictors of SN metastasis in cN0 patients, and these parameters could help anticipate metastasis of non-SN in SN-positive patients. Furthermore, in cN0 and SN metastasis-positive patients with low SUVmax2 and low ΔSUVmax%, the negative status of non-SN could be predicted with high probability (92.3%) by using a combination of SUVmax2 and ΔSUVmax% values.

Several clinicopathological factors have been described as predictors of SN metastasis in breast cancer.^13^ These factors include tumor size, lymphovascular invasion, HER2, ER, multifocality, age, and tumor grade. Furthermore, several studies identified clinicopathological predictors of non-SN metastasis such as primary tumor size, lymphovascular invasion, and SN status.^24^–^26^ Several nomograms have been developed to predict SN and non-SN metastasis: the MSKCC nomogram of prediction of metastasis to SN and non-SN utilizes primary tumor features such as tumor size, tumor grade, and lymphovascular invasion.^15^,^16^ In the present cohort, the MSKCC nomograms were confirmed to be useful for prediction of SN and non-SN metastasis. In these nomograms, some pathological parameters of a primary tumor can only be obtained from detailed postoperative pathological examination. Because SUVmax of the primary tumor was correlated with these pathological parameters and was able to be acquired before surgical examination, the measurement of the SUVmax may potentially be of clinical benefit.

In the present study, pT, ER, and Ly were independent predictors of SN metastasis, and tumor invasion size was significantly different between non-SN-metastasis-positive and non-SN-metastasis-negative groups. ER-positive cN0 cases showed significantly higher odds ratio of SN metastasis than ER-negative cN0 cases. Although this result appeared paradoxical, it is in agreement with findings of other large-scale studies.^15^

^18^F-FDG PET/CT was performed using the DTP method, and it was confirmed that the combination of SUVmax2 and ΔSUVmax% was useful to predict non-SN metastasis using preoperative features. This combination was also superior in sensitivity (94.7%) and NPV (92.3%) to SUVmax1, SUVmax2, and ΔSUVmax% alone, and to the combination of SUVmax1 and ΔSUVmax%. These results might support the idea that the combination of SUVmax and ΔSUVmax% represents a more biological characteristic of the tumor.

Given the findings of ACOSOG Z0011 and AMAROS trials, in cN0 and SN-positive patients, axillary radiotherapy could be chosen instead of ALND if further axillary treatment is needed.^14^,^27^ At present, the findings of additional non-SN may not be clinically useful. However, which subset of cN0 and SN-positive patients require axillary treatment is not fully clarified. If SUVmax parameters of PET/CT scans were shown to accurately predict SN and non-SN statuses, these results would open the way to further research to find the optimal axillary management in cN0 and SN-positive patients.

PET/CT scans are not performed routinely, and the SUVmax parameters are not introduced clinically for many patients. If SUVmax parameters are shown to be excellent for prediction of SN and/or non-SN metastasis and their utility is widely accepted, this method may be included as one preoperative diagnostic tool in the future.

The cost of ^18^F-FDG PET/CT is higher than the total cost of whole-body examinations, including MRI, bone scintigraphy, and abdominal ultrasonography, but ^18^F-FDG PET/CT appears to have superiority in that the diagnosis of both local and systemic status of a disease is possible in only 3 h. Therefore, the PET/CT was considered to be more convenient to the patients than the combination of other whole-body examinations.

Limitations of this study include its retrospective nature and that it was conducted in a single facility with a relatively small number of patients. Another prospective multicenter trial is needed to confirm the effectiveness of SUVmax and ΔSUVmax% in the prediction of SN and non-SN metastasis.

In conclusion, SUVmax of the primary tumor was a predictive factor of SN and/or non-SN metastasis in patients with cN0 breast cancer. Furthermore, it was possible to estimate non-SN metastasis negativity with a high probability by combining SUVmax2 and ΔSUVmax%. From these results arises the possibility of minimizing unnecessary ALND.

## Electronic supplementary material

Below is the link to the electronic supplementary material.

Distribution of probabilities of (A) SN metastasis and (B) non-SN metastasis, according to the MSKCC nomograms. (A) Median probabilities of SN metastasis 58.0% (11.0–98.0%) and 34.0% (0.0–93.0%) in groups A and B, respectively. (B) Median probabilities of non-SN metastasis 28.0% (11.0–68.0%) and 20.0% (9.0–77.0%) in groups C and D, respectively. Significant differences between group A and B (*P* < 0.0001) and between group C and D (*P* = 0.0296) (TIFF 547 kb)
