# Supplementary figures and images for: Utility of 18F-Fluorodeoxyglucose Positron Emission Tomography/Computed Tomography Fusion Imaging for Prediction of Metastasis to Sentinel and Nonsentinel Nodes in Patients with Clinically Node-Negative Breast Cancer
Source: Ann Surg Oncol. 2020 Mar 2;27(8):2698–710. doi: 10.1245/s10434-020-08269-0 (PMC7334280; doi:10.1245/s10434-020-08269-0)

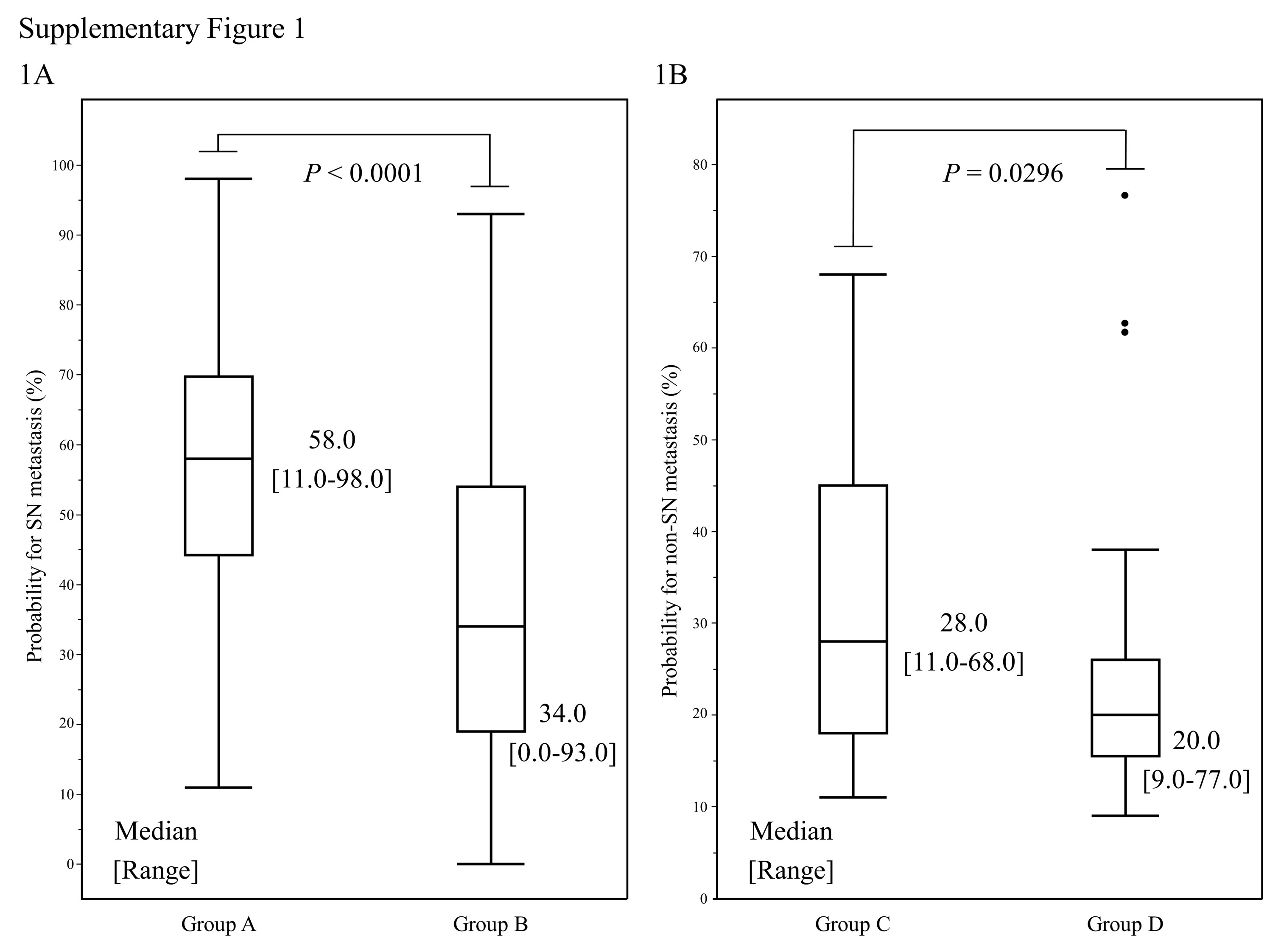

Supplement: Supplementary file 1 — Distribution of probabilities of (A) SN metastasis and (B) non-SN metastasis, according to the MSKCC nomograms. (A) Median probabilities of SN metastasis 58.0% (11.0–98.0%) and 34.0% (0.0–93.0%) in groups A and B, respectively. (B) Median probabilities of non-SN metastasis 28.0% (11.0–68.0%) and 20.0% (9.0–77.0%) in groups C and D, respectively. Significant differences between group A and B (P < 0.0001) and between group C and D (P = 0.0296) (TIFF 547 kb) [file 10434_2020_8269_MOESM1_ESM.tif]
